# Simultaneous placement of short implants (≤ 8 mm) versus standard length implants (≥ 10 mm) after sinus floor elevation in atrophic posterior maxillae: a systematic review and meta-analysis

**DOI:** 10.1186/s40729-022-00443-1

**Published:** 2022-10-05

**Authors:** Chenxi Tang, Qianhui Du, Jiaying Luo, Lin Peng

**Affiliations:** State Key Laboratory of Oral Diseases, Department of Implantology, West China Hospital of Stomatology, National Clinical Research Center for Oral Diseases, Sichuan University, Chengdu, China

**Keywords:** Dental implant, Survival rate, Marginal bone loss, Complication

## Abstract

**Purpose:**

The objective of this meta-analysis was to compare the clinical outcomes of using short implants (≤ 8 mm) inserted with osteotome sinus floor elevation (OSFE) and standard implants (≥ 10 mm) inserted with sinus floor elevation (SFE) in atrophic posterior maxillae with insufficient residual bone height (RBH).

**Methods:**

An electronic search was performed on PubMed, EMBASE, and the Cochrane Library from 1994 to July 2022, in combination with a manual search of references in relevant articles. Randomized controlled trials (RCTs) that compared the clinical results between short and standard implant placement with SFE were included. The primary outcomes were implant survival rate and marginal bone loss (MBL); the secondary outcome was complication rate.

**Results:**

Three RCTs were included, totaling 138 short and 156 standard implants. The results of the meta-analysis showed no significant differences between the short and standard implant groups in survival rate (RR = 1.02, 95% CI   0.96–1.08, *p* = 0.570), MBL (MD = − 0.13, 95% CI   − 0.32 to 0.07, *p* = 0.190) and complication rate (intra-surgical complication: RR = 1.14, 95% CI   0.46–2.83, *p* = 0.770; post-operative complication: RR = 1.34, 95% CI   0.71–2.55, *p* = 0.370).

**Conclusions:**

Using short implants (≤ 8 mm) combined with OSFE might be an alternative to standard implants (≥ 10 mm) with SFE when the RBH of the posterior maxilla is insufficient. Based on a short-term clinical observation, short implants with OSFE show good results in terms of survival rate, MBL, and complication incidence.

**Graphical Abstract:**

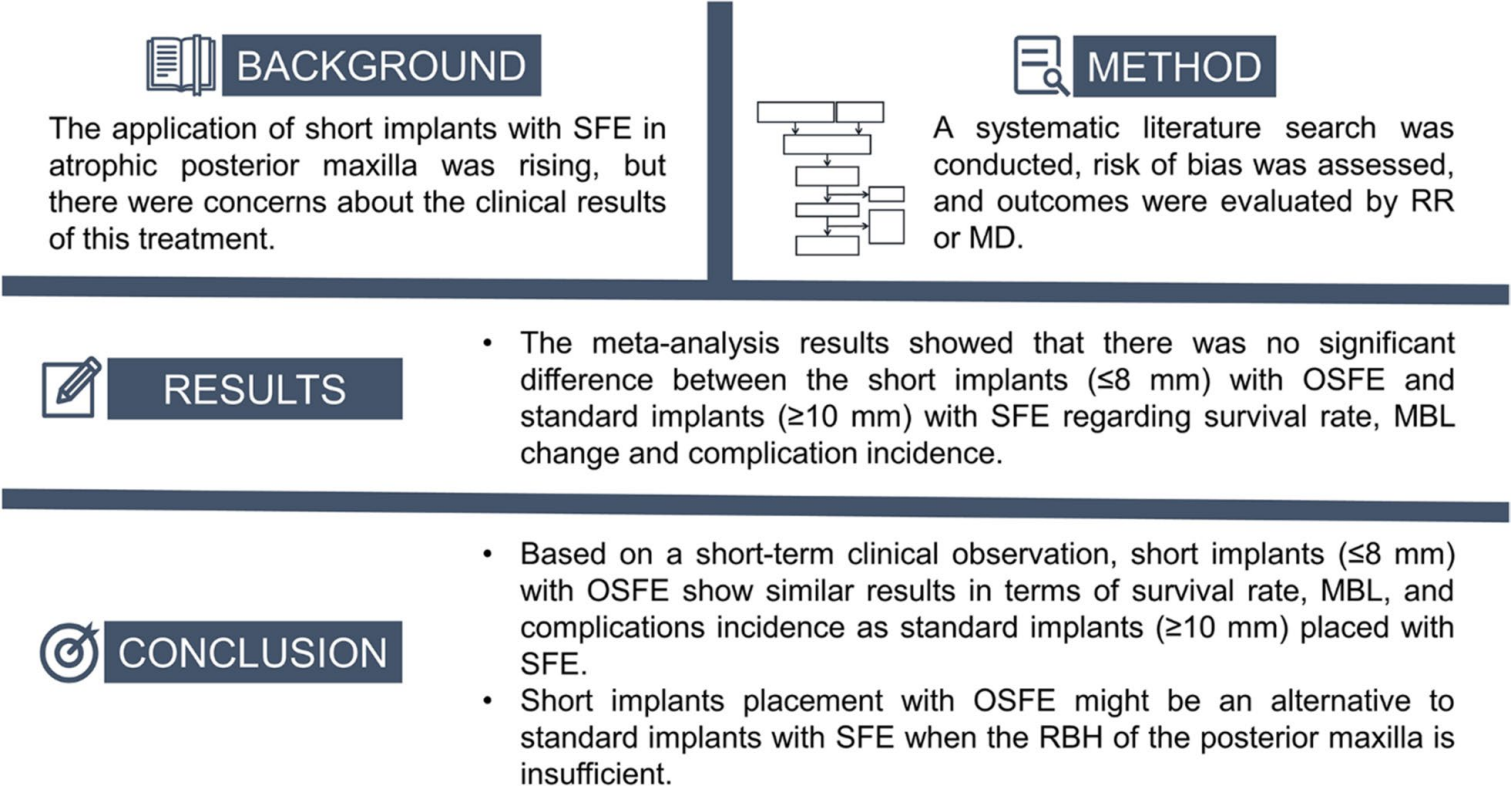

## Introduction

Dental implants have become a routine treatment for patients with edentulous jaw and dentition defects. After tooth loss, alveolar ridge resorption and maxillary sinus pneumatization result in poor bone quality and insufficient residual bone height (RBH) in the posterior maxilla [1]. When placing standard implants directly in an atrophic posterior maxilla, maxillary sinus floor elevation (SFE) was routinely used to increase bone volume [2]. Standard implants placed after SFE can achieve a high implant survival rate (94–100%) [3–5]. However, this procedure is expensive, time-consuming, and associated with high morbidity and invasive surgical operations [6].

Due to improvements in implant design and surface modification, short implants have become more popular in atrophic posterior maxillae than SFE. They are more acceptable to patients because of their low cost, faster surgical time, and less invasive and uncomplicated surgical procedures [7, 8]. Some authors consider implants with a length range of 7–10 mm to be “short” [9], while recent studies defined an implant with an intra-bony length of ≤ 8 mm to be a short implant [10]. In this study, implants with a length of ≤ 8 mm were considered as short implants [11]. Short implants in the posterior maxilla have a clinical survival rate comparable to standard implants [12–14]. A systematic review reported that using short implants (mean implant length, 6.56–8.20 mm) in the posterior maxilla could achieve acceptable clinical results (survival rate, 86.5–98.2%) with 5–10 year follow-up [15]. Nevertheless, short implants have a relatively high crown-to-implant (C/I) ratio and limited functional surface area, which are not conducive to osseointegration and stress distribution; thus, some researchers doubt the use of short implants in posterior maxillae with osteoporosis [16, 17].

In cases with insufficient available bone in the posterior maxillae, short implants are considered an effective alternative to standard implants with SFE. Several systematic reviews and meta-analyses recently showed that routine placement of short implants in the posterior maxilla could achieve similar clinical results as standard implants combined with SFE [18]. An umbrella review by Vetromilla et al. reported that short implants had similar implant survival rates, reduced marginal bone loss (MBL), and fewer biologic complication rates compared to longer implants with SFE [19]. In contrast, Cruz et al. reported that short implants have similar survival rates but fewer biological complications and MBL compared to longer implants with SFE; however, the risk of prosthetic complications of short implant placement is higher [20].

In the atrophic posterior maxilla, approximately 32% of second premolar positions, 73% of first molar positions, and 54% of second molar positions exhibit RBH < 5 mm; therefore, using short implants cannot avoid SFE in these areas [21]. Moreover, when the available bone height is < 3 mm, using standard implants denotes that most cases need lateral SFE (LSFE), which increases surgical trauma, expenses, and risk of postoperative complications while also challenging the surgeons’ technical abilities. Several clinical studies have begun using short implants combined with osteotome SFE (OSFE) to avoid these issues. These studies indicate that short implants with OSFE can achieve favorable clinical outcomes, which is an effective method to avoid standard implants placed with OSFE or LSFE for severely atrophic posterior maxillae [22, 23]. A systematic review stated that it was still unclear if short implants placed with OSFE have a lower or higher survival rate than standard implants combined with OSFE when the RBH was insufficient [24]. However, studies in this meta-analysis were observational studies, which cannot provide high-quality evidence compared with randomized controlled trials (RCTs). Several meta-analyses focused on whether there were differences in clinical outcomes between short implants without SFE and standard implants combined with SFE in atrophic posterior maxillae. Nevertheless, there was no relevant systematic review and meta-analysis based on RCTs comparing the clinical outcomes between short implants combined with OSFE and standard implants with OSFE or LSFE in the atrophic posterior maxilla.

Therefore, the primary purpose of this systematic review and meta-analysis was to compare the results of RCTs examining differences between short implants (≤ 8 mm) combined with OSFE and standard implants (≥ 10 mm) with OSFE/LSFE in the severely atrophic posterior maxilla. In addition, the null hypothesis is that the implant survival rate, MBL, and complications of short implants are comparable to those of standard implants.

## Materials and methods

This systematic review was conducted based on the basis of the Preferred Reporting Items for Systematic Reviews and Meta-Analysis. The protocol was registered in the PROSPERO database with the registration number CRD42022295859.

### Search strategy

An electronic systematic literature search was conducted using PubMed, Embase, and the Cochrane Library from 1994 to July 2022. The relevant articles’ references were used as secondary reference sources (manual search). The literature search strategy for the three electronic databases is presented in Table 1. The last search was performed on July 9, 2022. The main search terms were: “sinus floor augmentation,” “sinus floor elevation,” “sinus floor lift,” and “dental implants.”

**Table 1:** Search strategies

Database	Search strategy
PubMed	("Dental Implants"[MeSH Terms] OR "dental implant"[Title/Abstract]) AND ("sinus floor elevation"[Title/Abstract] OR "sinus floor lift"[Title/Abstract] OR "Sinus Floor Augmentation"[MeSH Terms])
Embase	('tooth implant'/exp OR 'dental implant*':ti,ab) AND ('sinus floor augmentation'/exp OR 'sinus floor elevation':ti,ab OR 'sinus floor lift':ti,ab)
Cochrane	(MeSH descriptor: [Dental Implants] OR ‘dental implant’:ti,ab,kw) AND (MeSH descriptor: [Sinus Floor Augmentation] OR ‘sinus floor elevation’:ti,ab,kw OR ‘sinus floor lift’:ti,ab,kw)

### Eligibility criteria

Relevant studies were identified based on the following inclusion criteria (PICOS): (a) participants: patients with insufficient RBH (< 8 mm) in the atrophic posterior maxilla, regardless of age, sex, country, and the number of teeth lost; (b) intervention: patients who underwent OSFE and short implant (≤ 8 mm) placement simultaneously with RBH of the inserted site was < 8 mm; (c) comparison: patients who underwent OSFE or LSFE and standard implant (≥ 10 mm) placement simultaneously when RBH of the inserted site was < 8 mm; (d) outcomes: the primary outcomes were implant survival rate and MBL and the secondary outcome was the complication rate; and (e) study: RCTs.

The exclusion criteria include: (a) cohort studies, case–control studies, cross-sectional studies, descriptive studies, case reports and systematic reviews; (b) studies involving animals; short or standard implants placed without SFE; (c) implants placed at the second stage of the procedure after SFE; the study was unable to collect data or had insufficient data.

Regarding the multiple publications of the same patient population, only the publication with the longest follow-up period was included.

### Study selection

EndNote20 software (Clarivate; Pennsylvania, Philadelphia, New York) was used to remove duplicate studies, and the remaining publications were screened manually. Two authors scanned all the titles and abstracts and conducted the study selection independently. For studies in which the title or abstract did not explicitly present the inclusion criteria and all eligible studies, full texts were obtained to determine whether the study fulfilled the PICOS. When differences arose regarding the selection of studies, a discussion was held and a third reviewer was engaged to reach a consensus.

### Data extraction

Two authors independently examined and extracted data from the entire texts of the included studies. The data included were: (a) the first name of the author; (b) study type; (c) publication date; (d) follow-up duration; (e) method of SFE; (f) implant system; (g) RBH; (h) the number of implants; and (i) primary and secondary outcomes.

### Quality assessment

Two authors investigated the risk of bias in the included studies independently. Disagreements were resolved through discussion. RCTs were evaluated using the Revised Cochrane risk of bias tool for randomized trials (RoB 2.0; Cochrane Collaboration; Mountain View, California, United States) [25]. When an RCT provided detailed data on all the parameters, the potential risk of bias was considered low; a study was considered to be of some concern if it lacked data on only one parameter and to have a high risk of bias if it failed to provide data on two or more parameters.

### Statistical analysis

The meta-analysis was conducted using Reviewer Manager 5.3 software (Cochrane Collaboration; Mountain View, California, United States). The risk ratio (RR) was used to evaluate dichotomous outcomes (implant survival rate and complications). Continuous outcome (MBL change) was assessed with the mean difference (MD) and 95% confidence intervals (CIs). Random-effect models were used; RR was calculated using the Mantel–Haenszel analysis; MD was calculated through inverse variance (*α* = 0.05). RR and MD values were considered significant when *p* < 0.050. Heterogeneity was evaluated using the Cochrane’s *Q* test and *I*^2^ statistic and was considered significant if *p* < 0.100 in the *Q* test. When there was significant heterogeneity, sensitivity analysis was performed in STATA 14.0 (StataCorp LLC; College Station, Texas, United States), and subgroup analysis was conducted in Reviewer Manager 5.3 software.

## Results

### Study selection

**Fig. 1:**
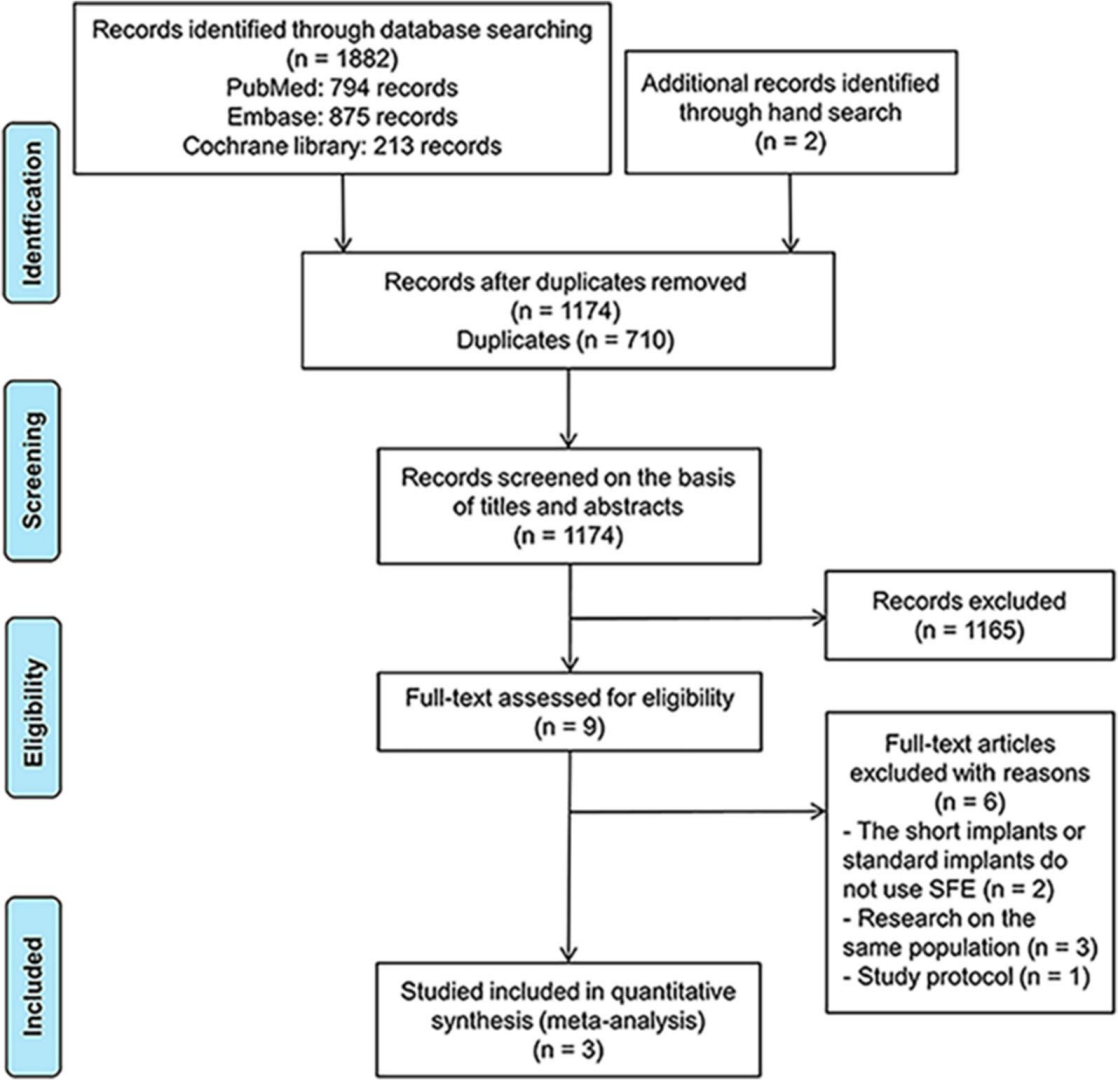
Flow diagram of the search strategy

The electronic database search identified 1882 articles (794 from PubMed, 875 from Embase, and 213 from Cochrane), and the manual search identified two articles (Fig. 1). After removing 710 duplicates, titles and abstracts of the remaining 1174 articles were screened. The full texts of nine potentially eligible articles were browsed; three studies that met the inclusion criteria were selected for the final analysis [26–28]. Figure 1 describes the reasons why studies were removed from the review.

### Study characteristics

This review included three RCTs [26–28], and Table 2 outlines their characteristics. The length of short implants ranged from 6.5 to 8 mm, and that of standard implants ranged from 10 to 16 mm. In the study by Shi et al., both the short and standard implant groups underwent OSFE [26], while in the other two studies, only the standard implant group underwent LSFE [27, 28]. The follow-up periods of the included studies were 3, 5 and 2 years, respectively. Finally, 138 and 156 implants were included in the short implant and standard implant groups, respectively.

**Table 2:** Characteristics of included studies

Author Publication	Study type	Implant groups	Number of patients, *n*	Implant number, *n*	Implant length, mm	Implant system	Failed implant, *n*
Shi et al. 2021	RCT	Short implants + OSFE	62	62	8	Straumann implants	1
		Standard implants + OSFE	70	70	10		0
Cannizzaro et al. 2013	RCT	Short implants + OSFE	20	38	8	Tapered Screw-Vent MP-1 HA Dual Transition Selective Surface	10
		Long implants + LSFE	20	44	10,13 or 16		5
Yu et al. 2016	RCT	Short implants + OSFE	20	38	6.5	Thommen dental implants	0
		Standard implants + LSFE	18	42	11 or 12.5		1

Author Publication	Implant groups	Survival rate (%)	MBL, mm	Intra-surgical complication, *n*	Post-surgical complication, *n*	RBH, mm	Follow-up, y
Shi et al. 2021	Short implants + OSFE	98.4	0.50 ± 0.30	7 sinus membrane perforation	21 peri-implant mucositis, 2 peri-implantitis	≥ 6, < 8	3
	Standard implants + OSFE	100	0.53 ± 0.28	6 sinus membrane perforation	13 peri-implant mucositis, 1 peri-implantitis		
Cannizzaro et al. 2013	Short implants + OSFE	97.4	0.41 ± 0.42	0	1 peri-implant bone loss, 1 peri-implantitis	3–6	5
	Long implants + LSFE	88.6	0.72 ± 0.41	2 sinus membrane perforation	1 abscess and 1 sinusitis, 1 peri-implant mucositis		
Yu et al. 2016	Short implants + OSFE	100	0.35 ± 0.60	2 sinus membrane perforation	4 nasal bleeding with postoperative headache	4–5	2
	Standard implants + LSFE	97.6	0.40 ± 0.71	1 sinus membrane perforation	1 abscess, 5 nasal bleeding		

*RCT* randomized controlled trial, *OSFE* osteotome sinus floor elevation, *LSFE* lateral sinus floor elevation, *RBH* residual bone height, *MBL* marginal bone loss

### Risk of bias

**Fig. 2:**
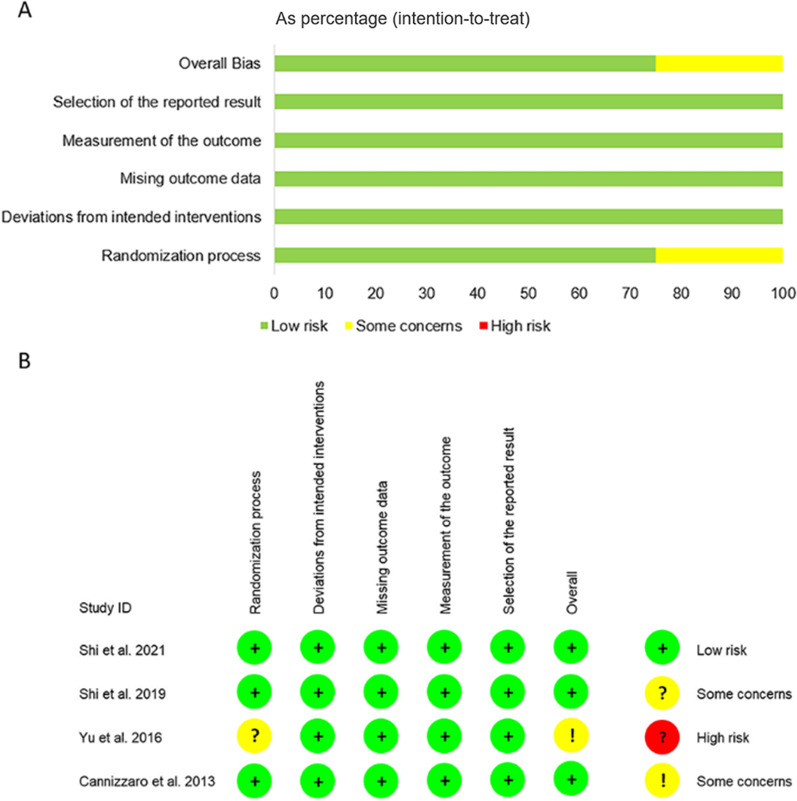
Risk of bias for included RCTs. **A** Each risk of bias item presented as percentages across the randomized controlled trials. **B** Each risk of bias item for each randomized controlled trial

RoB 2.0 was used to evaluate the included RCTs. Figure 2 shows the results of the risk of bias assessment. Shi et al. published certain results of the same population in their previous RCT study; therefore, a risk assessment of their previous study was also performed [26, 29]. Among the included RCTs, three exhibited a low risk of bias [26, 27, 29]; however, the study by Yu et al. was labeled as having some concerns due to its unclear random allocation method [28].

### Survival rate

**Fig. 3:**
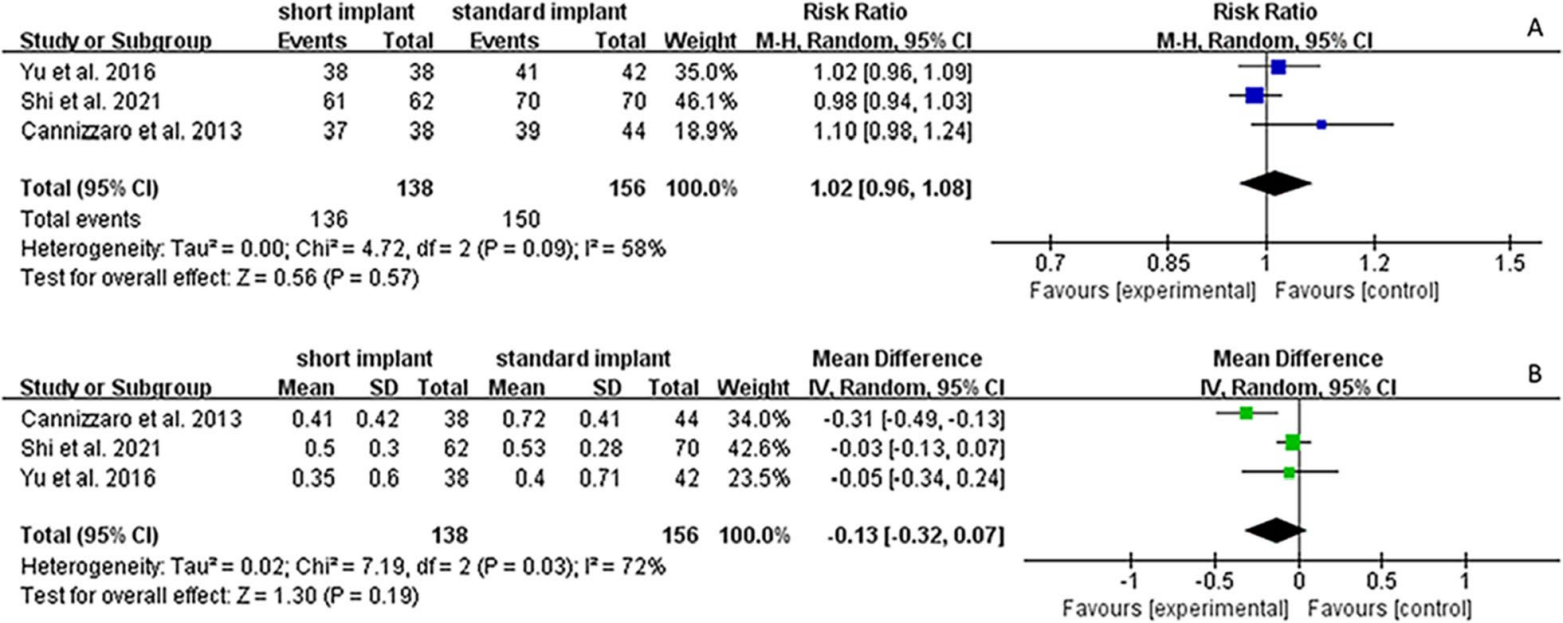
Forest plots for the survival rate and marginal bone loss of included studies. **A** Forest plot for risk ratio (RR) of failure between short implants with OSFE and standard implants with SFE in RCTs. **B** Forest plot for mean difference (MD) of marginal bone level changes between short implants with OSFE and standard implants with SFE. *CI* confidence interval

**Fig. 4:**
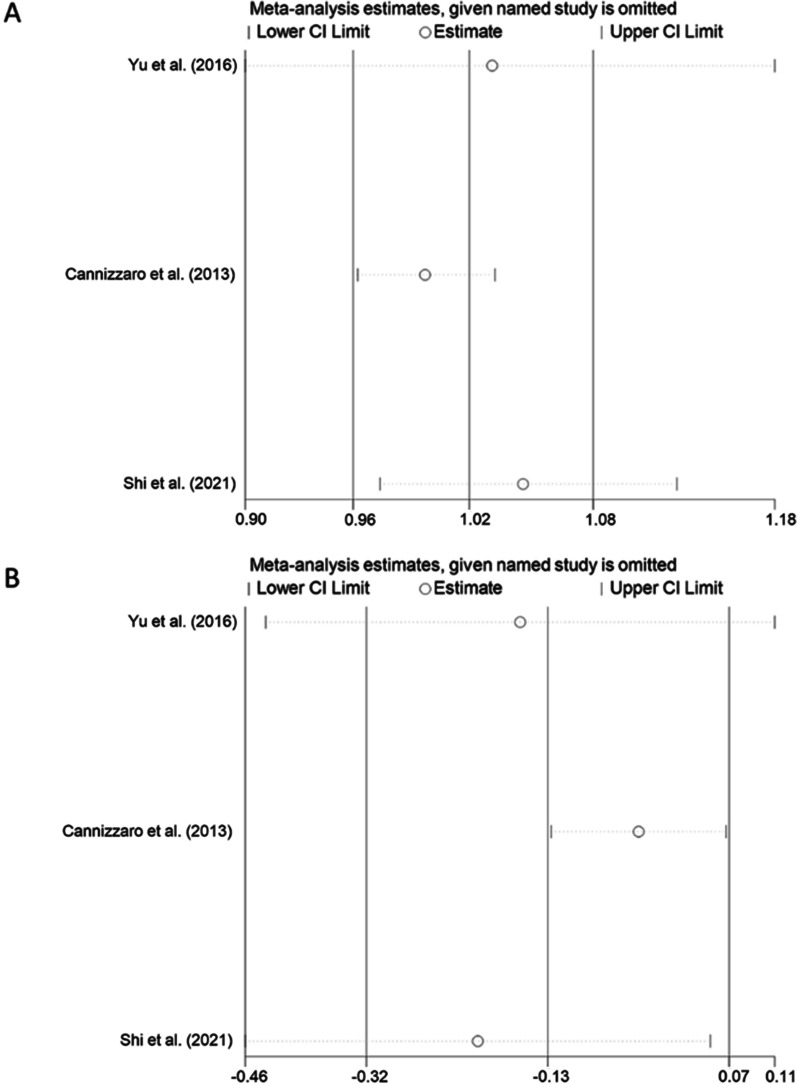
Sensitivity analysis for the survival rate and MBL of RCTs. Sensitivity analysis ascertained the effect of each study on the overall estimate by removing each study in the meta-analysis. **A** Sensitivity analysis plot for the heterogeneity survival rate, showing that after omitting any included study, the 95% CI contained 1, so the results remained table. **B** Sensitivity analysis for the heterogeneity of marginal bone level changes, showing that after omitting any included study, the 95% CI contained 0, so the results remained table. *CI* confidence interval

**Fig. 5:**
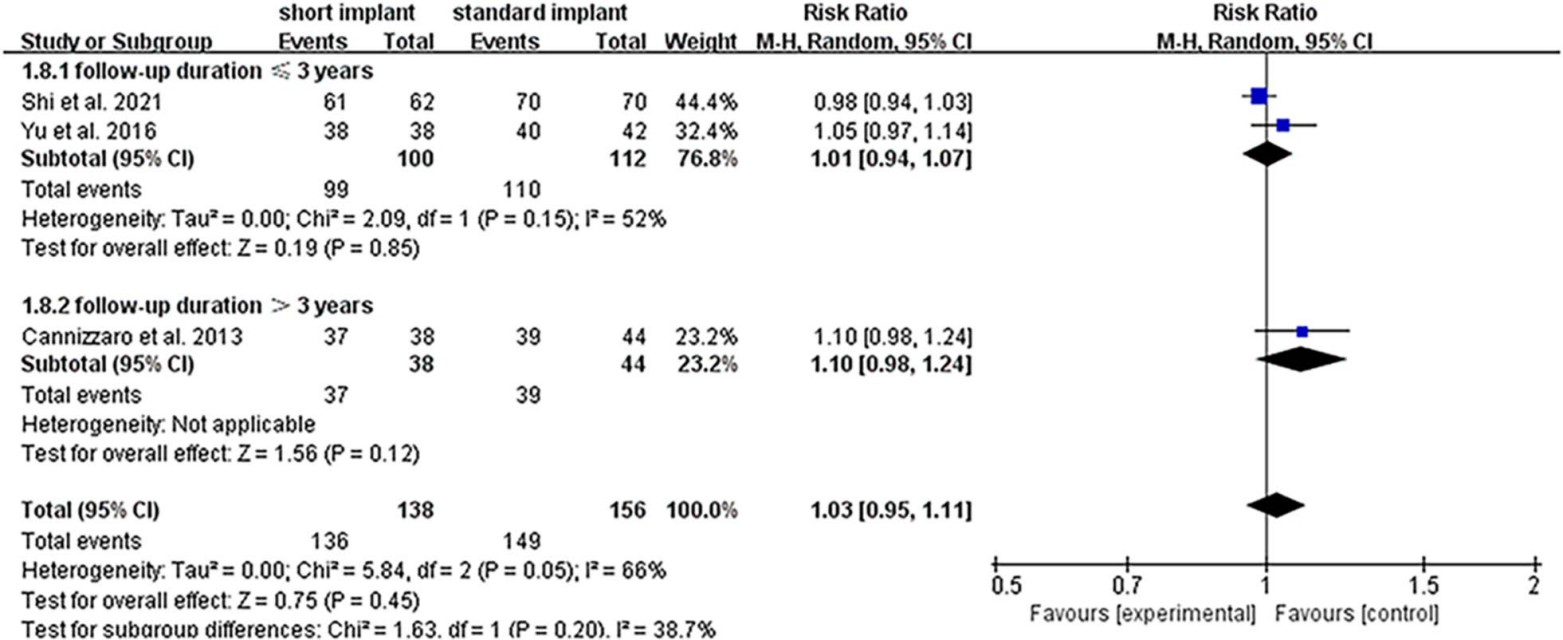
Subgroup analysis in follow-up duration. The *p* value of subgroup analysis was higher than the analysis of total studies, showing that the different follow-up durations might be a reason for heterogeneity

All included studies reported the implant survival rate. The overall survival rate of the included implants was 97.3%, and individual survival rates for short and standard implants were 98.6% and 96.2%, respectively. Figure 3A shows the meta-analysis result of the random-effect model for the survival rate of the included studies (*I*^2^ = 58%, *P* = 0.09, RR = 1.02, 95% CI 0.96–1.08, *p* = 0.57). Concerning implant survival rates, the 95% CI included the value 1, indicating no significant difference between the short implant group using OSFE and the standard implant group using SFE; however, the heterogeneity among RCTs was relatively high (*P* = 0.090). Sensitivity and subgroup analysis performed to determine the source of heterogeneity of RCTs, indicated that the results remained stable and that the heterogeneity may have been affected by different follow-up durations of the included studies, respectively (Figs. 4A and 5).

### MBL changes

**Fig. 6:**
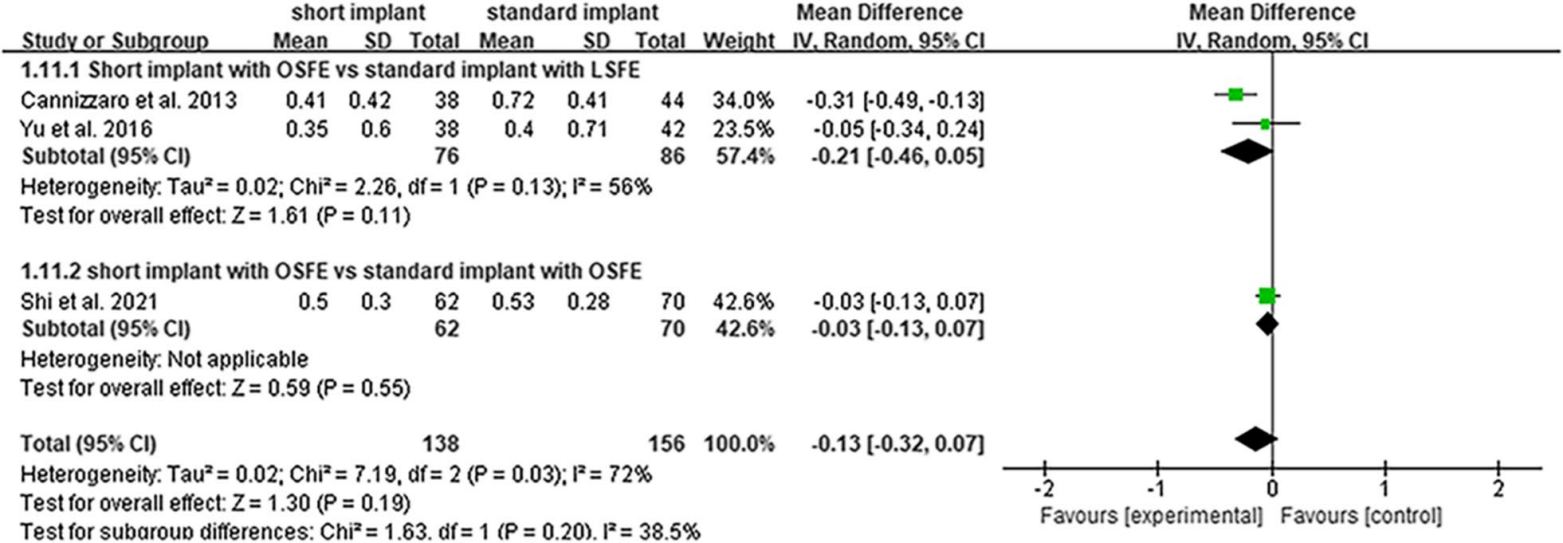
Subgroup analysis in sinus floor elevation methods of survival rate. The *p* value of subgroup analysis was higher than the analysis of total studies, showing that the different methods of sinus floor elevation might be a reason for heterogeneity. *OSFE* osteotome sinus floor elevation, *LSFE* lateral sinus floor elevation

The three included studies reported the results of MBL. As described, the mean MBL in the 2-year follow-up study by Yu et al. was 0.35 mm for the short implant group (6.5 mm) and 0.40 mm for the standard implant group (11 mm or 12.5 mm) [28]. In the 3-year follow-up study by Shi et al., the mean MBL was 0.50 mm and 0.53 mm in the short (8 mm) and standard implant (10 mm) groups, respectively [26]. In the 5-year follow-up study by Cannizzaro et al., the mean MBL was 0.41 mm and 0.72 mm in the short (8 mm) and standard implant groups (10 mm, 13 mm or 16 mm), respectively [27]. Although Shi et al. and Yu et al. did not show a significant difference in MBL changes between short and standard implants, (*p* = 0.897 and *p* = 0.751, respectively) [26, 28], Cannizzaro et al. showed significantly less MBL in short implants than in standard implants (*p* = 0.028) [27]. The meta-analysis results of the random-effect model (Fig. 3B), indicate no significant difference between them, as the 95% CI of MD included the value 0 (*I*^2^ = 72%, *P* = 0.03, MD = − 0.13, 95% CI   − 0.32–0.07, *p* = 0.190). However, the heterogeneity among the studies was significant. Sensitivity analysis and subgroup analysis were conducted to find out the heterogeneity of RCTs. The sensitivity analysis indicated that the results remained stable (Fig. 4B). Subgroup analysis indicated that the heterogeneity might be caused by different SFE methods within the included studies (Fig. 6).

### Complications

**Fig. 7:**
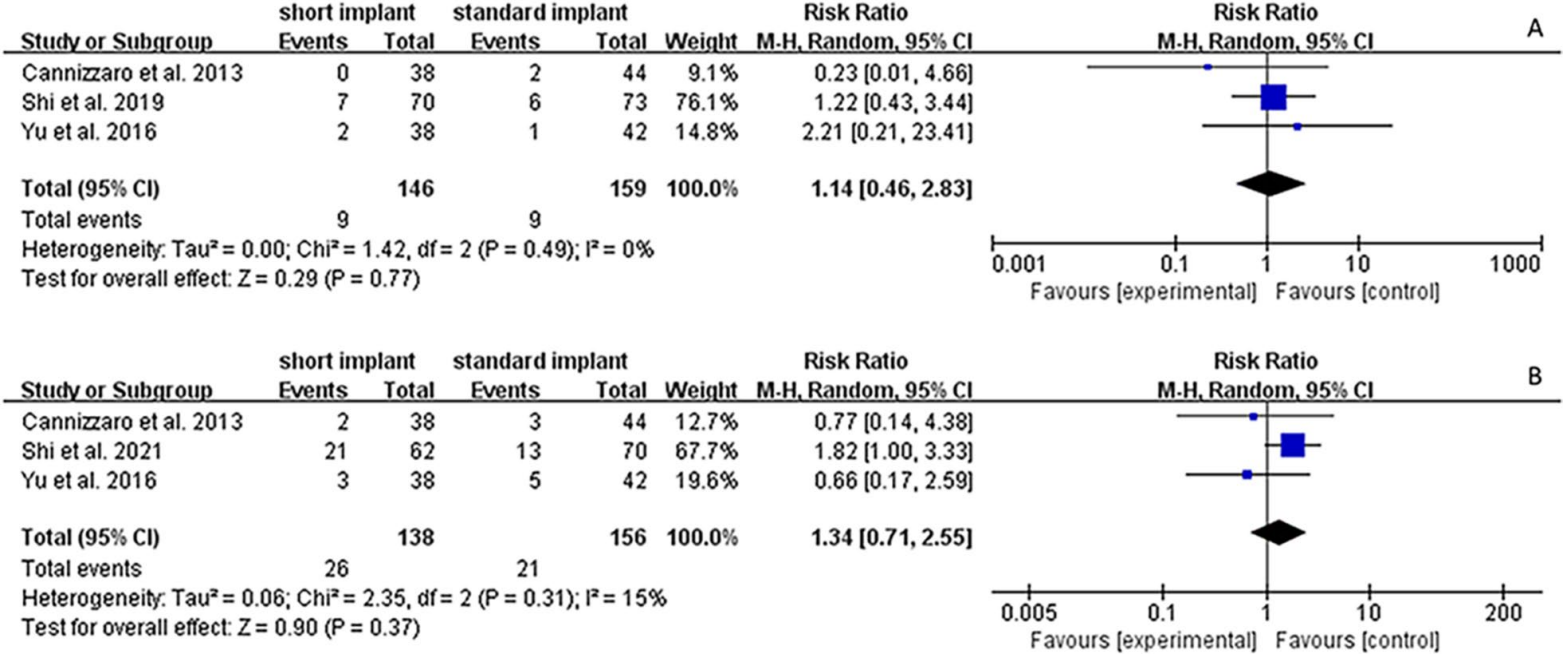
Forest plots for intra-surgical complication, and postoperative complication. **A** Forest plot for risk ratio (RR) of intra-surgical complication between short implants with OSFE and standard implants with SFE. **B** Forest plot for risk ratio (RR) of post-surgical complication between short implants with OSFE and standard implants with SFE. *CI* confidence interval

The three included studies discussed intra-operative and postoperative complications (Table 2). Sinus membrane perforation was the most prevalent intra-surgical complication. Of the 294 implants, 18 had mucosal perforation (nine cases in both short and standard implant groups). Cannizzaro et al. reported two perforation cases in the standard implant group but no perforation in the short implant group [27]. Yu et al. reported two perforation cases in the short implant group and one in the standard implant groups [28]. Shi et al. reported seven and six perforation cases in the short and standard implant groups, respectively [29]. Among the 294 implants, there was one case of peri-implant bone loss (0.34%), two of abscess (0.68%), one of maxillary sinusitis (0.34%) and nine of nasal bleeding with postoperative headache (3.06%) [26–28]. Peri-implantitis was the most common postoperative complication, with Shi et al. and Cannizzaro et al. reporting 21 cases of peri-implant mucositis (15.22%) and three of peri-implantitis (2.17%) in the short implant group, while 14 cases of peri-implant mucositis (8.97%) and one of peri-implantitis (0.64%) occurred in the standard implant group [26, 27]. A meta-analysis demonstrated the differences in complication rates between short and standard implants. Figure 7A, B shows the meta-analysis results of the random-effect model for intra-surgical complications (*I*^2^ = 0.0%, *P* = 0.49, RR = 1.14, 95% CI   0.46–2.83, *p* = 0.770) and post-surgical complications (*I*^2^ = 15%, *P* = 0.31, RR = 1.34, 95% CI   0.71–2.55, *p* = 0.370), respectively. The 95% CI of RR for both intra-surgical and post-operation complications included the value 1, indicating that no significant difference was found between the short and standard implant groups, and heterogeneity among the studies was relatively low.

## Discussion

The primary purpose of this systematic review was to compare the clinical outcomes of using short implants (≤ 8 mm) combined with OSFE and standard implants (≥ 10 mm) combined with SFE in the posterior atrophic maxilla of insufficient RBH (< 8 mm). The meta-analysis showed no significant difference between the short and standard implant groups regarding survival rate, MBL change, and complication incidence.

For implant survival rate of short (≤ 8 mm) and standard implants (≥ 10 mm), there was no significant difference between the two groups (RR = 1.02, 95% CI   0.96–1.08, *p* = 0.570), and both survival rates were relatively high (short implants: 97.0%, standard implants: 96.8%). The survival rate in this study is consistent with the results of the recently published systematic reviews. A systematic review by Lin et al., including one cohort study and 10 cross-sectional studies stated that regarding the survival rate at 1-year, healing period or 3-year loading, the differences between short implants (≤ 8 mm) and conventional implants (> 8 mm) both combined with transcrestal SFE were not significant (1 year: *I*^2^ = 0%, odds ratio [OR] = 1.04, 95% CI   0.55–1.96; healing period: *I*^2^ = 10%, OR = 0.74, 95% CI   0.28–1.97; 3-year loading: OR = 1.76, 95% CI   0.65–4.74, respectively) [24]. Another network meta-analysis by Al-Moraissi et al. showed that no significant difference was found in the survival rate between the short (4–8.5 mm) and long implant group (> 8 mm) when using OSFE in the posterior maxilla with RBH 4–8 mm (OR = 1.09, 95% CI   0.06–18.99) [30], which indicated that the length of the implant did not influence the survival rate. Some studies that assessed whether implant length influenced survival rate also had similar outcomes. Mijiritsky et al. showed that the survival rates of short (< 10 mm) and regular (≥ 10 mm) implants were 97% and 98.7%, respectively, and implant length was not a significant factor affecting implant survival during the first 2 years of function (*p* = 0.220) [31]. These results showed that short implants combined with OSFE was a feasible rehabilitation method for posterior maxillae with insufficient RBH and could achieve relatively high short- and long-term survival rates.

In our systematic review, two studies reported the reasons for failed implants, in which the short implant group used the OSFE method and the standard implant group used LSFE. Cannizzaro’s study showed that one short implant failed because of abutment looseness, and two standard implants were removed due to severe acute sinusitis that occurred 2 weeks after surgery [27]. Yu et al. reported that only one long implant failed due to abscess formation after surgery [28]. In published studies, the effect of surgical method on implant survival remains controversial. A 15-year retrospective study reported that when using OSFE or LSFE for atrophic maxillae, the implant survival rate was comparable (97.7% versus 97.3%) [32]. Another systematic review described a survival rate varying from 95.4% to 100% for the OSFE method in eight studies, while the survival rate of LSFE in 29 studies varied more widely from 75.57% to 100% [15]. Lundgren et al. stated that a lower survival rate of LSFE could result from the technique being utilized more frequently in cases with insufficient RBH, resulting in a higher risk of failure [33]. Several researchers agreed that OSFE was less traumatic than LSFE, and was a better choice in suitable situations [30, 33].

This meta-analysis included three studies that reported the implant MBL change, and no significant difference was found between the short and standard implant groups (MD = − 0.13, 95% CI   − 0.32–0.07, *p* = 0.190). Several clinical studies compared MBL changes between short and standard implant placement without SFE in the posterior maxilla and obtained different results. A systematic review found no significant difference in the MBL between short (≤ 8 mm) and standard implants (> 8 mm) without SFE (*p* = 0.240; RR = 1.35; 95% CI   0.82–2.22) [34]. Another 3-year follow-up multi-center RCT indicated that bone resorption of short implants (6 mm) was significantly less than standard implants (11 mm) without SFE (*p* < 0.050) [12]. Some researchers have commented on possible reasons why short implants affect MBL values. Yazicioglu et al. stated that short implants could change the stress distribution and increase the stress of the cortical and cancellous bone around the implants [35]. Overload and stress concentration had a greater impact on the bone level around the implant [36, 37]. In case of the same crown height, the short implants have a higher C/I than standard implants. Sotto-Maior et al. indicated that a high C/I ratio was an important factor that increased the stress concentration of short implants [38]. A 3-year follow-up prospective study showed that excessive bone loss and implant failure might occur when the anatomical C/I exceeded 3.1 or the clinical C/I exceeded 3.4 [17]. On the contrary, several studies stated high C/I ratio had no influence on bone resorption around the implant [39–41]. Moreover, Mezzomo et al. reported that regarding the short implants (≤ 10 mm), increased C/I ratios of short implant-supported single crowns did not affect MBL [42]. Other studies stated that compared to the implant length and the C/I ratio, the implant diameter was the critical factor affecting MBL. Bechara and Shimmlová et al. reported that the diameter of the implant had a greater correlation with MBL; wide implants facilitated the distribution of stress to the alveolar bone around the implant, thereby reducing stress concentration, and subsequently reducing MBL [43, 44]. These results indicated that for short implants, the C/I ratio might not influence MBL change; however, an appropriate range of C/I ratio and implant diameter should be considered to avoid the potential bone resorption risk of short implant application combined with OSFE.

In this systematic review, the meta-analysis results demonstrated no significant difference between the short and standard implant groups regarding intra-surgical (RR = 1.14, 95% CI   0.46–2.83, *p* = 0.770) and postoperative complications (RR = 1.34, 95% CI   0.71–2.55, *p* = 0.370). Sinus membrane perforation was the most common intra-surgical complication of SFE, and the perforation rate varied among different studies and techniques [45–48]. As Chen et al. and Călin et al. reported, sinus membrane perforation rates after OSFE were 0–10.8% and 6.28%, respectively [49, 50]. In addition, Cortes et al. and de Vicente et al. reported that the incidences of perforation with the LSFE method were 7.14% and 11.9%, respectively [51, 52]. However, whether perforation affects the implant survival rate remains unclear. A systematic review by Viña-Almunia et al. found that the implant survival rate in perforation cases after SFE was 88.6%, while that of non-perforation cases was 98% [53]. Another retrospective study demonstrated that the implant survival rate in the perforation and non-perforation groups were 100% and 95.5%, respectively [54]. The review of Díaz-Olivares LA et al. reported that there was no significant difference in implant survival rate between the perforation group (97.68%) and non-perforation group (98.88%) (*p* = 0.229) with appropriate management to perforation [55]. Perforation might not affect implant survival rate but it could cause postoperative complications [46, 56]. A systematic review applying 6–16 mm implants combined with OSFE reported that the most common postoperative complications were nasal bleeding (2.97%), followed by postoperative paroxysmal vertigo (2.17%), and the least common was postoperative infection (1.50%) [50]. The postoperative complication rate in our systematic review was consistent with previous studies. Several studies reported different results on whether the complication rate of short implant placement alone was less than the standard implant. A systematic review by Xu et al. reported that the incidence of biological complications of short implants (< 7 mm) and long implants (≥ 7 mm) were 15.8% and 41%, respectively, in which the former was much lower than the latter (RR: 0.38, 95% CI 0.27–0.54, *p* < 0.001) [57]. Another systematic review showed that the biological complications of ultra-short implants (≤ 6 mm) were significantly fewer than those of long implants (≥ 10 mm) based on a 1- and 3-year follow-up period (RR = 0.32, 95% CI   0.19–0.54, *p* = 0.040; RR = 0.28, 95% CI   0.19–0.43, *p* = 0.003, respectively), but showed no significant difference between them after a 5-year follow-up period (RR = 1.79, 95% CI   0.25–12.78, *p* = 0.600) [58]. In our systematic review, the follow-up periods of included studies were different, although the intra-surgical and post-surgical complication rates between short and standard implant groups showed no significant difference. Therefore, more studies with the same observation period should be conducted to obtain meaningful clinical result in the future.

In this systematic review, Shi et al. reported the implant stability quotient (ISQ), while Cannizzaro et al. showed that the ISQ value changes at several timepoints [27, 29]. As the property of the results in the two studies differed, we did not analyze the ISQ value. Implant stability is related to the survival rate, and a decrease in stability could increase the risk of implant failure [59]. Shi reported that ISQ values of the primary and secondary implant stability between the short and standard implant groups were not significantly different (primary stability, *p* = 0.470; secondary stability, *p* = 0.630) [29]. Lai et al. stated that the implant length, RBH, and bone type did not affect the ISQ value of the implant at the implant placement stage [60]. In addition, Pommer et al. stated that bone density seemed to be the essential factor affecting the implant primary stability, while the implant diameter and RBH have no effect when using the 5–6 mm short implants combined with OSFE [61]. These results indicated that short implant placement with OSFE in the atrophic maxilla could obtain high implant primary stability, which might contribute to the success of implant osteointegration.

The results of this systematic review and meta-analysis are limited by several factors that may have an effect. The number of included studies is limited, the follow-up duration in each study is relatively short and variable, and the evaluation indices of some included studies are insufficient. Therefore, additional long-term studies comparing more clinical outcomes of short implants (≤ 8 mm) with OSFE and standard implants (≥ 10 mm) with SFE in atrophic posterior maxillae are needed to draw more powerful conclusions.

## Conclusions

For patients with insufficient RBH in the atrophic posterior maxilla, short implants (≤ 8 mm) placed with OSFE could obtain similar clinical outcomes as standard implants (≥ 10 mm) placed with SFE. Based on a relatively short-term clinical observation, short implant placement with OSFE has a high implant survival rate, can obtain high implant stability, and demonstrates less MBL and fewer intra-surgical and post-surgical complications. This method might be an effective alternative to standard implant placement with SFE when the RBH of the posterior maxilla is insufficient.

## Data Availability

All data generated or analyzed during this study are included in this review.

## References

[1] Wagner F, Dvorak G, Nemec S, Pietschmann P, Figl M, Seemann R (2017). A principal components analysis: how pneumatization and edentulism contribute to maxillary atrophy. Oral Dis.

[2] Lucia S, Alessandro P, Giulia B, Giada F, Massimo DF, Daniele B (2022). The bone lid technique in lateral sinus lift: a systematic review and meta-analysis. Int J Implant Dent.

[3] Antonoglou GN, Stavropoulos A, Samara MD, Ioannidis A, Benic GI, Papageorgiou SN (2018). Clinical performance of dental implants following sinus floor augmentation: a systematic review and meta-analysis of clinical trials with at least 3 years of follow-up. Int J Oral Maxillofac Implants.

[4] Thoma DS, Zeltner M, Hüsler J, Hämmerle CH, Jung RE (2015). EAO Supplement Working Group 4—EAO CC 2015 short implants versus sinus lifting with longer implants to restore the posterior maxilla: a systematic review. Clin Oral Implants Res.

[5] Schiegnitz E, Kämmerer PW, Sagheb K, Wendt AJ, Pabst A, Al-Nawas B (2017). Impact of maxillary sinus augmentation on oral health-related quality of life. Int J Implant Dent.

[6] Esposito M, Felice P, Worthington HV (2014). Interventions for replacing missing teeth: augmentation procedures of the maxillary sinus. Cochrane Database Syst Rev.

[7] Schwartz SR (2020). Short implants: an answer to a challenging dilemma?. Dent Clin N Am.

[8] Jain N, Gulati M, Garg M, Pathak C (2016). Short implants: new horizon in implant dentistry. J Clin Diagn Res.

[9] Caramês J, Pinto AC, Caramês G, Francisco H, Fialho J, Marques D (2020). Survival rate of 1008 short dental implants with 21 months of average follow-up: a retrospective study. J Clin Med.

[10] Renouard F, Nisand D (2006). Impact of implant length and diameter on survival rates. Clin Oral Implants Res.

[11] Nizam N, Gürlek Ö, Kaval ME (2020). Extra-short implants with osteotome sinus floor elevation: a prospective clinical study. Int J Oral Maxillofac Implants.

[12] Zadeh HH, Guljé F, Palmer PJ, Abrahamsson I, Chen S, Mahallati R (2018). Marginal bone level and survival of short and standard-length implants after 3 years: an open multi-center randomized controlled clinical trial. Clin Oral Implants Res.

[13] Tolentino da Rosa de Souza P, BinhameAlbini Martini M, Reis Azevedo-Alanis L (2018). Do short implants have similar survival rates compared to standard implants in posterior single crown? A systematic review and meta-analysis. Clin Implant Dent Relat Res.

[14] Perelli M, Abundo R, Corrente G, Saccone C (2012). Short (5 and 7 mm long) porous implants in the posterior atrophic maxilla: a 5-year report of a prospective single-cohort study. Eur J Oral Implantol.

[15] Corbella S, Taschieri S, Del Fabbro M (2015). Long-term outcomes for the treatment of atrophic posterior maxilla: a systematic review of literature. Clin Implant Dent Relat Res.

[16] Ramos Verri F, Santiago Junior JF, de Faria Almeida DA, de Oliveira GB, de Souza Batista VE, Marques Honório H (2015). Biomechanical influence of crown-to-implant ratio on stress distribution over internal hexagon short implant: 3-D finite element analysis with statistical test. J Biomech.

[17] Malchiodi L, Cucchi A, Ghensi P, Consonni D, Nocini PF (2014). Influence of crown-implant ratio on implant success rates and crestal bone levels: a 36-month follow-up prospective study. Clin Oral Implants Res.

[18] Fan T, Li Y, Deng WW, Wu T, Zhang W (2017). Short implants (5 to 8 mm) versus longer implants (>8 mm) with sinus lifting in atrophic posterior maxilla: a meta-analysis of RCTs. Clin Implant Dent Relat Res.

[19] Vetromilla BM, Mazzetti T, Pereira-Cenci T (2021). Short versus standard implants associated with sinus floor elevation: an umbrella review of meta-analyses of multiple outcomes. J Prosthet Dent.

[20] Cruz RS, Lemos CAA, Batista VES, Oliveira H, Gomes JML, Pellizzer EP (2018). Short implants versus longer implants with maxillary sinus lift. A systematic review and meta-analysis. Braz Oral Res.

[21] Kopecka D, Simunek A, Brazda T, Rota M, Slezak R, Capek L (2012). Relationship between subsinus bone height and bone volume requirements for dental implants: a human radiographic study. Int J Oral Maxillofac Implants.

[22] Teng M, Liang X, Yuan Q, Nie J, Ye J, Cheng Q (2013). The inlay osteotome sinus augmentation technique for placing short implants simultaneously with reduced crestal bone height. A short-term follow-up. Clin Implant Dent Relat Res.

[23] Nedir R, Nurdin N, Abi Najm S, El Hage M, Bischof M (2017). Short implants placed with or without grafting into atrophic sinuses: the 5-year results of a prospective randomized controlled study. Clin Oral Implants Res.

[24] Lin ZZ, Jiao YQ, Ye ZY, Wang GG, Ding X (2021). The survival rate of transcrestal sinus floor elevation combined with short implants: a systematic review and meta-analysis of observational studies. Int J Implant Dent.

[25] Zeng X, Zhang Y, Kwong JS, Zhang C, Li S, Sun F (2015). The methodological quality assessment tools for preclinical and clinical studies, systematic review and meta-analysis, and clinical practice guideline: a systematic review. J Evid Based Med.

[26] Shi JY, Lai YR, Qian SJ, Qiao SC, Tonetti MS, Lai HC (2021). Clinical, radiographic and economic evaluation of short-6-mm implants and longer implants combined with osteotome sinus floor elevation in moderately atrophic maxillae: a 3-year randomized clinical trial. J Clin Periodontol.

[27] Cannizzaro G, Felice P, Minciarelli AF, Leone M, Viola P, Esposito M (2013). Early implant loading in the atrophic posterior maxilla: 1-stage lateral versus crestal sinus lift and 8 mm hydroxyapatite-coated implants. A 5-year randomised controlled trial. Eur J Oral Implantol.

[28] Yu H, Wang X, Qiu L (2017). Outcomes of 6.5-mm hydrophilic implants and long implants placed with lateral sinus floor elevation in the atrophic posterior maxilla: a prospective, randomized controlled clinical comparison. Clin Implant Dent Relat Res.

[29] Shi JY, Li Y, Qiao SC, Gu YX, Xiong YY, Lai HC (2019). Short versus longer implants with osteotome sinus floor elevation for moderately atrophic posterior maxillae: a 1-year randomized clinical trial. J Clin Periodontol.

[30] Al-Moraissi EA, Altairi NH, Abotaleb B, Al-Iryani G, Halboub E, Alakhali MS (2019). What is the most effective rehabilitation method for posterior maxillas with 4 to 8 mm of residual alveolar bone height below the maxillary sinus with implant-supported prostheses? A frequentist network meta-analysis. J Oral Maxillofac Surg.

[31] Mijiritsky E, Mazor Z, Lorean A, Levin L (2013). Implant diameter and length influence on survival: interim results during the first 2 years of function of implants by a single manufacturer. Implant Dent.

[32] Tetsch J, Tetsch P, Lysek DA (2010). Long-term results after lateral and osteotome technique sinus floor elevation: a retrospective analysis of 2190 implants over a time period of 15 years. Clin Oral Implants Res.

[33] Lundgren S, Cricchio G, Hallman M, Jungner M, Rasmusson L, Sennerby L (2017). Sinus floor elevation procedures to enable implant placement and integration: techniques, biological aspects and clinical outcomes. Periodontol 2000.

[34] Lemos CA, Ferro-Alves ML, Okamoto R, Mendonça MR, Pellizzer EP (2016). Short dental implants versus standard dental implants placed in the posterior jaws: a systematic review and meta-analysis. J Dent.

[35] Yazicioglu D, Bayram B, Oguz Y, Cinar D, Uckan S (2016). Stress distribution on short implants at maxillary posterior alveolar bone model with different bone-to-implant contact ratio: finite element analysis. J Oral Implantol.

[36] Quirynen M, Naert I, van Steenberghe D (1992). Fixture design and overload influence marginal bone loss and fixture success in the Brånemark system. Clin Oral Implants Res.

[37] Sheridan RA, Decker AM, Plonka AB, Wang HL (2016). The role of occlusion in implant therapy: a comprehensive updated review. Implant Dent.

[38] Sotto-Maior BS, Senna PM, da Silva WJ, Rocha EP, Del Bel Cury AA (2012). Influence of crown-to-implant ratio, retention system, restorative material, and occlusal loading on stress concentrations in single short implants. Int J Oral Maxillofac Implants.

[39] Blanes RJ (2009). To what extent does the crown-implant ratio affect the survival and complications of implant-supported reconstructions? A systematic review. Clin Oral Implants Res.

[40] Ramaglia L, Di Spirito F, Sirignano M, La Rocca M, Esposito U, Sbordone L (2019). A 5-year longitudinal cohort study on crown to implant ratio effect on marginal bone level in single implants. Clin Implant Dent Relat Res.

[41] Malchiodi L, Giacomazzi E, Cucchi A, Ricciotti G, Caricasulo R, Bertossi D (2019). Relationship between crestal bone levels and crown-to-implant ratio of ultra-short implants with a microrough surface: a prospective study with 48 months of follow-up. J Oral Implantol.

[42] Mezzomo LA, Miller R, Triches D, Alonso F, Shinkai RS (2014). Meta-analysis of single crowns supported by short (<10 mm) implants in the posterior region. J Clin Periodontol.

[43] Bechara S, Nimčenko T, Kubilius R (2017). The efficacy of short (6 mm) dental implants with a novel thread design. Stomatologija.

[44] Anitua E, Tapia R, Luzuriaga F, Orive G (2010). Influence of implant length, diameter, and geometry on stress distribution: a finite element analysis. Int J Periodontics Restor Dent.

[45] Danesh-Sani SA, Loomer PM, Wallace SS (2016). A comprehensive clinical review of maxillary sinus floor elevation: anatomy, techniques, biomaterials and complications. Br J Oral Maxillofac Surg.

[46] Schwartz-Arad D, Herzberg R, Dolev E (2004). The prevalence of surgical complications of the sinus graft procedure and their impact on implant survival. J Periodontol.

[47] Stacchi C, Andolsek F, Berton F, Perinetti G, Navarra CO, Di Lenarda R (2017). Intraoperative complications during sinus floor elevation with lateral approach: a systematic review. Int J Oral Maxillofac Implants.

[48] Jordi C, Mukaddam K, Lambrecht JT, Kühl S (2018). Membrane perforation rate in lateral maxillary sinus floor augmentation using conventional rotating instruments and piezoelectric device—a meta-analysis. Int J Implant Dent.

[49] Chen MH, Shi JY (2018). Clinical and radiological outcomes of implants in osteotome sinus floor elevation with and without grafting: a systematic review and a meta-analysis. J Prosthodont.

[50] Călin C, Petre A, Drafta S (2014). Osteotome-mediated sinus floor elevation: a systematic review and meta-analysis. Int J Oral Maxillofac Implants.

[51] de Vicente JC, Hernández-Vallejo G, Braña-Abascal P, Peña I (2010). Maxillary sinus augmentation with autologous bone harvested from the lateral maxillary wall combined with bovine-derived hydroxyapatite: clinical and histologic observations. Clin Oral Implants Res.

[52] Cortes AR, Pinheiro LR, Cavalcanti MG, Arita ES, Tamimi F (2015). Sinus floor bone failures in maxillary sinus floor augmentation: a case–control study. Clin Implant Dent Relat Res.

[53] Viña-Almunia J, Peñarrocha-Diago M, Peñarrocha-Diago M (2009). Influence of perforation of the sinus membrane on the survival rate of implants placed after direct sinus lift. Literature update. Med Oral Patol Oral Cir Bucal.

[54] Froum SJ, Khouly I, Favero G, Cho SC (2013). Effect of maxillary sinus membrane perforation on vital bone formation and implant survival: a retrospective study. J Periodontol.

[55] Díaz-Olivares LA, Cortés-Bretón Brinkmann J, Martínez-Rodríguez N, Martínez-González JM, López-Quiles J, Leco-Berrocal I (2021). Management of Schneiderian membrane perforations during maxillary sinus floor augmentation with lateral approach in relation to subsequent implant survival rates: a systematic review and meta-analysis. Int J Implant Dent.

[56] Kim JS, Choi SM, Yoon JH, Lee EJ, Yoon J, Kwon SH (2019). What affects postoperative sinusitis and implant failure after dental implant: a meta-analysis. Otolaryngol Head Neck Surg.

[57] Xu X, Huang J, Fu X, Kuang Y, Yue H, Song J (2020). Short implants versus longer implants in the posterior alveolar region after an observation period of at least five years: a systematic review and meta-analysis. J Dent.

[58] Ravidà A, Wang IC, Barootchi S, Askar H, Tavelli L, Gargallo-Albiol J (2019). Meta-analysis of randomized clinical trials comparing clinical and patient-reported outcomes between extra-short (≤6 mm) and longer (≥10 mm) implants. J Clin Periodontol.

[59] Andersson P, Pagliani L, Verrocchi D, Volpe S, Sahlin H, Sennerby L (2019). Factors influencing resonance frequency analysis (RFA) measurements and 5-year survival of neoss dental implants. Int J Dent.

[60] Lai HC, Zhang ZY, Wang F, Zhuang LF, Liu X (2008). Resonance frequency analysis of stability on ITI implants with osteotome sinus floor elevation technique without grafting: a 5-month prospective study. Clin Oral Implants Res.

[61] Pommer B, Hof M, Fädler A, Gahleitner A, Watzek G, Watzak G (2014). Primary implant stability in the atrophic sinus floor of human cadaver maxillae: impact of residual ridge height, bone density, and implant diameter. Clin Oral Implants Res.

